# Micronutrient levels and their effects on the prognosis of visceral leishmaniasis treatment, a prospective cohort study

**DOI:** 10.1186/s12879-020-05615-1

**Published:** 2020-11-19

**Authors:** Berhanu Elfu Feleke, Teferi Elfu Feleke

**Affiliations:** 1 Department of Epidemiology and Biostatistics, University of Bahir Dar, Bahir Dar, Ethiopia; 2 Department of pediatrics and child health, Wolkite University, Butajira, Ethiopia; 3 General hospital, Wolkite, Ethiopia

**Keywords:** Micronutrients, Kalazar, Visceral leishmaniasis, HIV, Ethiopia

## Abstract

**Background:**

Micronutrients are minerals and vitamins and they are essential for normal physiological activities. The objectives of the study were to describe the progress and determinants of micronutrient levels and to assess the effects of micronutrients in the treatment outcome of kalazar.

**Methods:**

A prospective cohort study design was used. The data were collected using patient interviews, measuring anthropometric indicators, and collecting laboratory samples. The blood samples were collected at five different periods during the leishmaniasis treatments: before starting anti-leishmaniasis treatments, in the first week, in the second week, in the third week, and in the 4th week of anti-leishmaniasis treatments. Descriptive statistics were used to describe the profile of patients and to compare the treatment success rate. The generalized estimating equation was used to identify the determinants of serum micronutrients.

**Results:**

The mean age of the patients were 32.88 years [SD (standard deviation) ±15.95]. Male constitute 62.3% of the patients and problematic alcohol use was present in 11.5% of the patients. The serum zinc level of visceral leishmaniasis patients was affected by alcohol (B − 2.7 [95% CI: − 4.01 - -1.5]), DDS (B 9.75 [95% CI: 7.71–11.79]), family size (B -1.63 [95% CI: − 2.68 - -0.58]), HIV (B -2.95 [95% CI: − 4.97 - -0.92]), and sex (B − 1.28 [95% CI: − 2.5 - -0.07]). The serum iron level of visceral leishmaniasis patients was affected by alcohol (B 7.6 [95% CI: 5.86–9.35]), family size (B -5.14 [95% CI: − 7.01 - -3.28]), malaria (B -12.69 [95% CI: − 14.53 - -10.87]), *Hookworm* (− 4.48 [− 6.82 - -2.14]*)*, chronic diseases (B -7.44 [95% CI: − 9.75 - -5.13]), and HIV (B -5.51 [95% CI: − 8.23 - -2.78]). The serum selenium level of visceral leishmaniasis patient was affected by HIV (B -18.1 [95% CI: − 20.63 - -15.58]) and family size (B -11.36 [95% CI: − 13.02 - -9.7]). The iodine level of visceral leishmaniasis patient was affected by HIV (B -38.02 [95% CI: − 41.98 - -34.06]), DDS (B 25 .84 [95% CI: 22.57–29.1]), smoking (B -12.34 [95% CI: − 15.98 - -8.7]), chronic illness (B -5.14 [95% CI: − 7.82 - -2.46]), and regular physical exercise (B 5.82 [95% CI: 0.39–11.26]). The serum vitamin D level of visceral leishmaniasis patient was affected by HIV (B -9.43 [95% CI: − 10.92 - -7.94]), DDS (B 16.24 [95% CI: 14.89–17.58]), malaria (B -0.61 [95% CI: − 3.37 - -3.37]), and family size (B -1.15 [95% CI: − 2.03 - -0.28]). The serum vitamin A level of visceral leishmaniasis patient was affected by residence (B 0.81 [95% CI: 0.08–1.54]), BMI (B 1.52 [95% CI: 0.42–2.6]), DDS (B 1.62 [95% CI: 0.36–2.88]), family size (B -5.03 [95% CI: − 5.83 - -4.22]), HIV (B -2.89 [95% CI: − 4.44 - -1.34]),MUAC (B 0.86 [95% CI: 0.52–1.21]), and age (B 0.09 [95% CI: 0.07–0.12]).

**Conclusion:**

The micronutrient levels of visceral leishmaniasis patients were significantly lower. The anti-leishmaniasis treatment did not increase the serum micronutrient level of the patients.

## Background

Leishmaniasis is a group of vector born disease caused by the *leishmania* species. The three forms of leishmaniasis are cutaneous leishmaniasis, mucocutaneous leishmaniasis, and visceral leishmaniasis [1]. The severest form of the disease is visceral leishmaniasis and every year, it affects around 90, 000 people globally [2, 3]. It was reported from Bangladesh, Brazil, Ethiopia, India, South Sudan, and Sudan [4–6]. In the Amhara region, 5000 visceral leishmaniasis patients were reported annually [7, 8].

Micronutrients are minerals and vitamins that are required in a small amount, and they are essential for normal physiological activities. They serve as co-factors for many important metabolic enzymes. They enhance the function of the immune system and regulate gene transcription [9]. Disease conditions, especially inflammatory disease decrease the micronutrient levels [10]. Visceral leishmaniasis is among the disease condition that decrease the patient’s micronutrient level [11, 12]. The serum micronutrient concentration can be affected by chronic diseases, BMI, smoking, DDS (dietary diversification score), physical activity, intestinal parasites, MUAC (mid-upper arm circumference), HIV, and ethnicity [13–26].

The impacts of visceral leishmaniasis and micronutrient deficiency were not limited to the patients and their families; it also affects the socio-economic development of the nations [27–29]. Evidence on micronutrient level of visceral leishmaniasis patient was scarce and this research work conducted to answer the following objectives during visceral leishmaniasis treatment

- Describe the progress of micronutrient levels during visceral leishmaniasis treatment.
- Identify the determinants of micronutrient levels.
- To assess the effects of micronutrient levels on the treatment outcome.

## Methods

### Study design

A prospective cohort study design was used.

### Settings

The study was conducted among visceral leishmaniasis patients taking their treatment in the Amhara national regional state leishmaniasis treatment centers. The five leishmaniasis treatment centers of the region are Felegehiwote referral hospital, Gondar University hospital, Metema hospital, Addis Zemen health center, and Abderafi health center. From these treatment centers, more than 5000 incident visceral leishmaniasis patients was reported annually [7, 8]. The data were collected from August 2016–May 2019.

### Participants

The target population for this study was adult visceral leishmaniasis patients receiving their treatment from the five treatment centers of the regional state. Visceral leishmaniasis patients fulfilling the world health organization case definition that means, a person with chronic fever, splenomegaly, and weight loss with parasitologically confirmed results were included. Visceral leishmaniasis patients with incomplete records were excluded from the study.

### Variables

The stool sample was collected to adjust for *hookworm*, which significantly affects the serum iron level. From each visceral leishmaniasis patient, one gram stool sample was collected in 10 ml SAF (sodium acetate-acetic acid-formalin solution). To see the presence of intestinal parasitic infection using concentration technique [30]. The blood samples were collected at five repeated times in a month: before starting anti-leishmaniasis treatments, in the first week, in the second week, in the third week, and in the 4th week of anti-leishmaniasis treatments. At each spot, five-milliliter blood sample was collected from each visceral leishmaniasis patient following standard operating procedures to measure the serum zinc, iron, vitamin A, vitamin D, and selenium level. Urine samples were collected simultaneously with blood samples to measure their iodine levels. High-performance liquid chromatography was used to measure the serum vitamin A level of the patient [31], atomic absorption spectrophotometer was used to measure the serum zinc and selenium levels [32, 33], serum iron level was measured using Cobas 6000 (Roche kits German) instruments (Set 2015; analytics 2014), mini Vitek Immune Diagnostic Assay System (VIDAS) machine was used to measure the serum vitamin D level and urine iodine level was measured using the Sandell Kolthoff reaction. Vitamin A, Iron, and Zinc were measured using micrograms per deciliter (*mcg/dl*); Iodine was measured using micrograms per liter (*mcg/l*); Selenium and Vitamin D were measured using Nanograms per milliliter (*ng/ml*). Strict quality control measures were implemented during each laboratory procedure. CAGE tool was used to detect problematic alcohol use [34], Dietary diversification score (DDS) was measured using the world health organization (WHO) recommendations [35]. An International physical activity questionnaire (IPAQ) was used to measure regular physical activity [36]. Treatment success was declared if the patients become symptom-free and the absence of *leishmania* parasites in microscopy.

### Data source

The data were collected using patient interview, measuring anthropometric indicators, collecting blood, urine, and stool samples. The interview and anthropometric indicators were collected by clinical nurses.

### Bias

Probability sampling was used to select the study participants that decrease the selection bias. To decrease information bias: Training was given for data collectors and supervisors, the whole data collection procedures were closely supervised, data were cleaned and cheeked for errors before data entry.

### Study size

The sample size was calculated using Epi-info software with the assumption of a 95% confidence interval, 90% power, HIV positive visceral leishmaniasis patients to HIV negative visceral leishmaniasis patients proportion of 1:2, risk ratio of 1.2 (HIV decreases the micronutrient level by 20%) and 15% loss to follow up rate; finally giving 465 HIV positive visceral leishmaniasis patients and 930 HIV negative visceral leishmaniasis patients. A systematic random sampling technique was used to select both HIV positive and HIV negative visceral leishmaniasis patients.

### Quantitative variables

Quantitative variables were checked for the outlier. Their measures of central tendency and measure of dispersion were reported after checking the correct assumption.

### Statistical methods

Data were entered into the computer using Epi-info software [37] and transported to SPSS for the analysis [38]. Descriptive statistics were used to describe the profile of patients and to compare the treatment success rate of visceral leishmaniasis patients. Generalized estimating equations (GEE, Autoregressive correlation matrix) were used to identify the determinants of serum micronutrients [39].

Ethical clearance was obtained from Bahir Dar University College of Medicine and Health Sciences ethical review committee. Permission was obtained from the Amhara national regional state health bureau and each treatment center. Written informed consent was obtained from each leishmaniasis patient before recruitment. Visceral leishmaniasis patients with abnormal laboratory findings were referred to the curative care segment of the hospital. The confidentiality of the data was kept at each level. Study participant’s right to withdraw from the research was respected at any point.

## Results

Totally, 1309 visceral leishmaniasis patients were followed, giving for a response rate of 94%; 32 patients did not volunteer to participate, the medical records of 31 VL (visceral leishmaniasis) patients were incomplete and 23 patients have died. Most of the study participants were included from Gondar university hospital (305), followed by Felegehiwote referral hospital (298), Metema hospital (268), Addis zemen health center (224), and Abderafi health center (214). The mean age of study participants was 32.88 years [SD ±15.95 years]. Male constitute 62.3% of study participants and problematic alcohol use was observed in 11.5% of the patients (Table 1).

**Table 1:** Population profile of the study participants (*n* = 1309)

Variables		Frequency	Percentage
Sex	Male	816	62.3
	Female	493	37.7
Resident	Rural	838	64
	Urban	471	36
Dietary diversification score	≥6	253	19.3
	3–5	332	25.4
	0–2	724	55.3
Problematic alcohol use	Present	150	11.5
	Absent	1159	88.5
Family size	> 4	669	51.1
	≤4	640	48.9
Other chronic illness	Present	150	11.5
	Absent	1159	88.5
Smoking	Yes	187	14.3
	No	1122	85.7
Malaria co-infection	Present	831	63.5
	Absent	478	36.5
HIV	Positive	420	32.1
	Negative	889	67.9
Hookworm	Infected	530	40.5
	Not infected	779	59.5
Body mass index	< 18.5	1100	84
	≥18.5	209	16

### Interpretations

Problematic alcohol use decreases the serum zinc level by 2.7 micrograms per deciliter (*mcg/dl*). Female kalazar patients had 1.28 *mcg/dl* less zinc level than males. High dietary diversification increases the serum zinc level by 9.75 *mcg/dl*. High family size decreases the serum zinc level by 1.63 *mcg/dl*. The serum zinc level of HIV positive visceral leishmaniasis patients was 2.95 *mcg/dl* less than HIV negative visceral leishmaniasis patients. The anti-leishmaniasis treatment did not increase the serum zinc level of the patients.

Problematic alcohol use increases the serum iron level of the patients by 7.6 *mcg/dl*. Chronic illness decreases the serum iron level of the patients by 7.44 *mcg/dl*. Malaria co-infection decreases the serum iron level of the patients by 12.69 *mcg/dl*. *Hookworm* infection decreases the serum iron level of visceral leishmaniasis patients by 4.48 *mcg/dl*. High family size decreases the serum iron level of the patients by 5.14 *mcg/dl*. HIV infection decreases the serum iron level of the patients by 5.54 *mcg/dl*. The serum iron level of patient increase by 0.11*mcq/dl* per a year increase in the patient age. The serum iron level of the patient increase by 0.75 *mcq/dl* per a centimeter increase in the MUAC of the patient. The anti-leishmaniasis treatment increases the serum iron level of the patient by 0.67 *mcg/dl*.

HIV positive visceral leishmaniasis patient had 18.1 *ng/ml* less serum selenium level than HIV negative visceral leishmaniasis patients. High family size decreases the serum selenium level of the patients by 11.36 *ng/ml*. The anti-leishmaniasis treatment increases the serum selenium level by 3.04 *ng/ml*.

Malaria decreases the iodine level of visceral leishmaniasis patients by 3.78 *mcg/l*. High DDS increases the iodine level of the patients by 25.84 *mcg/l*. Smoking decreases the iodine level of the patients by 12.34 *mcg/l*. HIV decreases the iodine level of visceral leishmaniasis patients by 38.02 *mcg/l*. Chronic illness decreases the iodine level of visceral leishmaniasis patients by 5.14 *mcg/l*. The anti-leishmaniasis treatment increases the iodine level of patients by 13.67 *mcg/l*.

Problematic alcohol use decreases the serum vitamin A level of visceral leishmaniasis patients by 1.09 *mcg/dl*. Chronic illness decreases the serum vitamin A level of the patients by 2.56 *mcg/dl*. Urban residence increases the serum vitamin A level of the patient by 0.81 *mcq/dl*. High DDS increases the serum vitamin A level of the patient by 1.62 *mcg/dl*. Malaria co-infection decreases the serum vitamin A level of the patient by 4.8 *mcg/dl*. High family size decreases the serum vitamin A level of visceral leishmaniasis patients by 5.03 *mcg/dl*. HIV infection decreases the serum vitamin A level of the patients by 2.89 *mcg/dl*. A centimeter increase in the MUAC of visceral leishmaniasis patients increases the serum vitamin A level by 0.86 *mcg/dl*. The anti-leishmaniasis treatment did not increase the serum vitamin A level of the patient.

A unit increase in the BMI of visceral leishmaniasis patients increases the serum vitamin D level by 1.52 *ng/ml*. High DDS increases the serum vitamin D level of the patients by 16.24 *ng/ml*. Malaria decreases the serum vitamin D level of the patients by 0.61 *ng/ml*. In the presence of *hookworm* infection, the serum vitamin D level of visceral leishmaniasis patients decreased by 3.94 *ng/ml*. High family size decreases the serum vitamin D level of the patients by 1.15 *ng/ml*. HIV co-infection decreases the serum vitamin D level of the patient by 9.43 *ng/ml*. The anti-leishmaniasis treatment did not increase the serum vitamin D level of visceral leishmaniasis patients (Table 2).

**Table 2:** Predictors of micronutrient level in visceral leishmaniasis patients (*n =* 1309)

Dependent Variables	Predictors	β [95% CI β]	***P***-value
Serum zinc level	Problematic alcohol use	-2.7[−4.01 - -1.5]	< 0.01
	Female sex	-1.28 [−2.5 - -0.07]	0.04
	DDS	9.75 [7.71–11.79]	< 0.01
	High family size	−1.63 [−2.68 - -0.58]	< 0.01
	HIV	−2.95 [−4.97 - -0.92]	< 0.01
	Age	−0.043 [−0.08 - -0.01]	0.01
	Anti-leishmaniasis treatments	0.09 [−0.3–0.48]	0.66
Iron	Alcohol	7.6 [5.86–9.35]	< 0.01
	Chronic diseases	−7.44 [−9.75 - -5.13]	0.01
	Malaria	−12.69 [−14.53 - -10.87]	< 0.01
	Hookworm	−4.48 [−6.82 - -2.14]	< 0.01
	High family size	−5.14 [−7.01 - -3.28]	< 0.01
	HIV	−5.51 [−8.23 - -2.78]	< 0.01
	Age	0.11 [0.07–0.15]	< 0.01
	MUAC	0.75 [0.21–1.29]	< 0.01
	Anti-leishmaniasis treatments	0.67 [0.08–1.27]	0.02
Serum Selenium	HIV	−18.1 [−20.63 - -15.58]	< 0.01
	High family size	−11.36 [− 13.02 - -9.7]	< 0.01
	Anti-leishmaniasis treatments	3.04 [2.32–3.76]	< 0.01
Iodine	Malaria	−3.78 [−6.16 - -1.39]	< 0.01
	DDS	25 .84 [22.57–29.1]	< 0.01
	Smoking	−12.34 [− 15.98 - -8.7]	< 0.01
	HIV	−38.02 [−41.98 - -34.06]	< 0.01
	Chronic illness	−5.14 [−7.82 - -2.46]	< 0.01
	Regular physical exercise	5.82 [0.39–11.26]	0.04
	Anti-leishmaniasis treatments	13.67 [13.15–14.2]	< 0.01
Vitamin A	Problematic alcohol use	−1.09 [− 2.01 - -0.17]	0.02
	Chronic illness	−2.56 [−3.53 - -1.59]	< 0.01
	Urban residence	0.81 [0.08–1.54]	0.03
	DDS	1.62 [0.36–2.88]	0.01
	Malaria	−4.8 [−5.91 - -3.85]	< 0.01
	High family size	−5.03 [− 5.83 - -4.22]	< 0.01
	HIV	−2.89 [−4.44 - -1.34]	< 0.01
	Age	0.09 [0.07–0.12]	< 0.01
	MUAC	0.86 [0.52–1.21]	< 0.01
	Anti-leishmaniasis treatments	−0.3 [− 0.62 - -0.17]	0.06
Vitamin D	BMI	1.52 [0.42–2.6]	< 0.01
	DDS	16.24 [14.89–17.58]	< 0.01
	Malaria	−0.61 [−3.37 - -3.37]	< 0.01
	*Hookworm*	−3.94 [−5.46 - -2.41]	< 0.01
	High family size	−1.15 [−2.03 - -0.28]	< 0.01
	HIV	−9.43 [− 10.92 - -7.94]	< 0.01
	Age	0.03 [0.001–0.06]	0.04
	Anti-leishmaniasis treatments	−0.94 [−1.25 - -0.63]	< 0.01

The micronutrient level directly affects treatment outcome of visceral leishmaniasis; especially the treatment outcome was not successful if the serum zinc, iron, vitamin A and vitamin D levels were lower than the first quartile. The overall treatment success rate of visceral leishmaniasis treatment was 84.7% [95% CI: 82.77 - 86.67%] (Tables 3, 4).

**Table 3:** Micronutrient level versus treatment outcomes

Micronutrient		Treatment outcome				RR^**a**^ [95% CI]
		Successful		Not successful		
		Frequency	%	Frequency	%	
Zinc (*mcg/dl*)	≤58	211	16.1	135	10.3	0.66 [0.6–0.72]
	59–98	387	29.6	36	2.8	0.98 [0.95–1.02]
	> 99	511	39	29	2.2	Reference group
Iodine (*mcg/l*)	≤113	224	17.1	109	8.3	0.69 [0.64–0.75]
	114–147	247	18.9	72	5.5	0.79 [0.75–0.84]
	≥148	638	48.7	19	1.5	Reference group
Iron (*mcg/dl*)	≤46	321	24.5	200	15.3	0.61 [0.75–0.85]
	47–48	146	11.2	0	0	0.99 [0.97–1]
	≥49	642	49	0	0	Reference group
Selenium (*ng/ml*)	≤84	264	20.2	68	5.2	0.88 [0.83–0.93]
	85–105	245	18.7	71	5.4	0.85 [0.8–0.91]
	≥106	600	45.8	61	4.7	Reference group
Vitamin A (*mcg/dl*)	≤16	233	17.8	136	10.4	0.64 [0.59–0.69]
	17–31	209	16	56	4.3	0.8 [0.75–0.85]
	≥32	667	51	8	0.6	Reference group
Vitamin D (*ng/ml*)	≤15	281	21.5	112	8.6	0.63 [0.59–0.69]
	16–27	219	16.7	27	2.1	0.79 [0.75–0.85]
	≥28	609	46.5	61	4.7	Reference group

^a^*RR* risk ratio

**Table 4:** The levels of micronutrients at each week of anti-leishmaniasis treatments

Micronutrients	Before treatments		At 1st week		At 2nd week		At 3rd week		At 4th week		***P***-value	Normal reference range	
	Mean	SD	Mean	SD	Mean	SD	Mean	SD	Mean	SD		Lower value	Upper value
Zinc in (*mcg/dl*)	86.43	25.80	91.31	18.98	98.37	11.28	93.94	17.85	85.56	23.69	0.66	70	125
Iodine in (*mcg/l*)	85.21	43.81	86.71	27.49	94.63	24.85	110.98	28.75	141.44	35.52	< 0.01	150	249
Iron in (*mcg/dl*)	63.61	38.10	58.61	26.04	53.92	22.37	58.72	22.80	66.92	35.35	0.02	60	170
Selenium in (*ng/ml*)	92.00	49.30	93.46	32.83	101.76	29.64	101.61	27.08	103.14	27.39	0.01	70	150
Vitamin A in (*mcg/dl*)	35.45	21.73	38.44	17.19	32.89	19.81	36.35	26.26	34.99	18.70	0.06	15	60
Vitamin D in (*ng/ml*)	34.84	19.49	32.55	17.39	33.86	21.72	31.30	16.31	30.79	17.87	< 0.01	20	40

## Discussion

Problematic alcohol use decreases the serum zinc level by 2.7 *mcg/dl,* and the serum vitamin A level by 1.09 *mcg/dl*. This finding was in line with previous research outputs [40, 41]. This is because alcohol interferes with the absorption and metabolism of zinc [42]. However, alcohol increases the serum iron level of visceral leishmaniasis patients by 7.6 *mcg/dl*. This is because alcohol increases the absorption of iron from the intestine [43].

A high dietary diversification score increases the serum zinc level of leishmaniasis patients by 9.75 *mcg/dl*, the iodine level by 25.84 *mcg/l*, the serum vitamin D level by *16.24 ng/ml*, and the serum vitamin A level by 1.62 *mcg/dl*. This finding agrees with previous work [44]. This is because high dietary diversification score increases access to enough quality and quantity of micronutrients [45].

A high family size decreases the serum zinc level of the patients by 1.63 *mcg/dl*, the serum iron level by 5.14 *mcg/dl*, the serum zinc level by 11.36 *ng/dl*, the serum vitamin A level by 5.03 *mcg/dl*, the serum vitamin D level of the patients by 1.15 *ng/ml*. This finding was in line with previous researches work [41, 46, 47]. This is due to the sharing of the limited micronutrient-rich foods to the unbalanced household family members [48].

The serum zinc level of HIV positive visceral leishmaniasis patient was 2.95 *mcg/dl* less than HIV negative visceral leishmaniasis patients, HIV positive visceral leishmaniasis patients had 5.54 *mcg/dl* less serum iron level than HIV negative visceral leishmaniasis patients, HIV positive visceral leishmaniasis patient had 18.1 *ng/dl* less serum selenium level than HIV negative visceral leishmaniasis patients, HIV decreases the iodine level of visceral leishmaniasis patients by 38.02 *mcg/l*, the serum vitamin A level of visceral leishmaniasis patients by 2.89 *mcg/dl*, the serum vitamin D level by 9.43 *ng/ml*. This finding agrees with previous research findings [21, 49]. This is due to the reason that HIV infection reduced the intake of food and absorption and increased utilization and loss of micronutrients [50].

Chronic illness decreases the serum iron level of visceral leishmaniasis patients by 7.44 *mcg/dl*, the iodine level by *5.14 mcg/l*, and the serum vitamin A level by 2.56 *mcg/dl*. This finding agrees with the 2019 published research work [51]. This is because the homeostasis of micronutrients, especially iron will be disturbed by chronic illnesses [52].

Malaria co-infection decreases the serum iron level of visceral leishmaniasis patients by 12.69 *mcg/dl*, the iodine level by *3.78 mcg/l*, the serum vitamin A level by 4.8 *mcg/dl* and the serum vitamin D level by *0.61 ng/ml*. This finding was in line with previously published works [53–55]. This is due to the ingestion of the nutrients by the parasites, decreases the intake from the host (anorexia), and increases the execration of the nutrients [56–58].

*Hookworm* infection decreases the serum iron level of visceral leishmaniasis patients by 4.48 *mcg/dl*; the serum vitamin D level by 3.94 *ng/ml*. This finding agrees with previous research outputs [59]. This is due to ingestion of nutrients by the parasites [60].

Per a year increase in the age of the patient, the serum iron level increases by 0.11 *mcg/dl*. This finding agrees with other’s scholars work [61]. This is due to the fact that serum iron decreasing factors like chronic diseases and other unhealthy lifestyles were prevalent in the older age [62].

Per a centimeter increase in the MUAC of the patient, the serum iron level increases by 0.75 *mcg/dl* and the serum vitamin A level by 0.86 *mcg/dl*. This finding was in line with previously published work [63]. This is due to the reason that, higher MUAC groups have good nutritional support [64].

Smoking decreases the iodine level of visceral leishmaniasis patients by 12.34 *mcg/l*. This finding agrees with previous scholar’s work [65]. This is due to the effect of smoking in disturbing the iodine metabolism [66, 67].

Leishmaniasis patients in the urban area had 0.81 *mcg/dl* higher serum vitamin A level than the rural patients. This finding agrees with finding from Nepal [68]. This is because of the higher awareness of the urban population about vitamin A [69].

A unit increase in the BMI of visceral leishmaniasis patients increases the serum vitamin D level by 1.52 *ng/ml*. This finding disagrees with finding from Norway [70]. This might be due to the cultural difference between the two populations.

The serum zinc level of females was 1.28 *mcg/dl* less than male. This finding agrees with previous literature [71]. This is because women lose their serum zinc level during their pregnancy and menstruation [72].

The anti-leishmaniasis treatment did not increase the serum zinc, vitamin A, vitamin D, or iron level of the patients. It increases the serum selenium level by 3.04 *ng/ml* and the iodine level by 13.67 *mcg/l*.

The overall treatment success rate of visceral leishmaniasis treatment was 84.7% [95% CI: 82.77 - 86.67%]. A systematic review and meta-analysis estimate also supports this finding [73].

Low serum zinc level decreases the treatment outcome of visceral leishmaniasis. This finding was in line with finding from India [12]. This is due to the effects of zinc in the immune system of the patients [74].

Higher patient iron level increases the treatment success rate of visceral leishmaniasis. This finding supports the results of previously published work [75]. This is due to the crucial role of iron in red blood cell production that is used to transport essential substances, including the anti-leishmaniasis drugs [76].

Higher serum vitamin A and Vitamin D level favors good treatment outcome in visceral leishmaniasis. This finding agrees with previous researchers outputs [77–79]. This indicates that administering the anti-leishmaniasis treatment alone will not yield a favorable treatment outcome in visceral leishmaniasis patients.

Possible limitation of this study was a failure to address all the vitamins and minerals status of visceral leishmaniasis patients, but since practically it is very difficult to address all of them this study gives the baseline evidence on main vitamins and mineral levels.

## Conclusion

The serum micronutrient levels of visceral leishmaniasis patients were low. Problematic alcohol use affects the serum zinc, iron, vitamin A levels. DDS affects the serum zinc, iodine, vitamin A, and vitamin D level. Family size affects the serum zinc, iron, selenium, vitamin A, and vitamin D levels. HIV infection affects the serum zinc, iron, selenium, iodine, vitamin A, and vitamin D levels. Anti-leishmaniasis drug slightly increases the serum iodine and selenium levels, but it doesn’t increase the serum iron, zinc, vitamin A, and vitamin D levels. The serum levels of zinc, iron, vitamin A, and vitamin D significantly affect the treatment outcomes of visceral leishmaniasis.

### Recommendation

The visceral leishmaniasis treatment guideline should incorporate supplementing the micronutrients as part of anti-leishmaniasis intervention.

## Data Availability

The datasets used and/or analyzed during the current study are available from the corresponding author on reasonable request.

## Abbreviations

B: Beta coefficient
BMI: Body mass index
CI: Confidence interval
DDS: Dietary diversification score
GEE: Generalized estimating eqs.
HIV: Human immune deficiency virus
MCG/DL: Micrograms per deciliter
Mg/dl: Milligram per deciliter
MUAC: Mid upper arm circumference
Ng/dl: Nanogram per deciliter
SD: Standard deviation
VL: Visceral leishmaniasis
WHO: World health organization
